# HMGA1 is a Prognostic Biomarker and Correlated with Glycolysis in Lung Adenocarcinoma

**DOI:** 10.7150/jca.89056

**Published:** 2024-03-25

**Authors:** Yu Ma, XiaoYu Ma, BuLin Du, XueNa Li, YaMing Li

**Affiliations:** 1 Department of Nuclear Medicine, The First Hospital of China Medical University, 110001, Liaoning Province, China.; 2 Departments of Gastrointestinal Endoscopy, The First Hospital of China Medical University, Shenyang 110001, Liaoning Province, China.

**Keywords:** lung adenocarcinoma, glycolysis, HMGA1, TCGA, bioinformatics

## Abstract

**Purpose:** Lung cancer is one of the leading causes with high morbidity and mortality. High mobility group A1 (HMGA1) protein participates in the process of tumorigenesis. This study seeks to explore the specific role of HMGA1 in prognostic value based on The Cancer Genome Atlas (TCGA) database of Lung adenocarcinoma (LUAD) and glycolysis progression in LUAD cells.

**Patients and Methods:** In this research, we compared HMGA1 mRNA expression between tumor tissues and normal samples and evaluated the correlations with clinical characteristics in LUAD patients based on the data of TCGA database. The survival outcome with overall survival (OS), disease-specific survival (DSS) and clinicopathologic characteristics associated were performed using the Kaplan-Meier method and Cox regression. In addition, gene-set enrichment analysis (GSEA) was carried out to explore the biological pathways that related to HMGA1. Cell experiments including cell proliferation assay and glycolysis proteins were performed with A549 and H1299 cells.

**Results:** Our results revealed that HMGA1 mRNA expression was higher in LUAD tissues than in normal tissues. Increased HMGA1 expression in LUAD was associated with Gender (p<0.01), Pathologic stage I&II vs stage III&IV (p<0.001), T1&T2 vs T3&T4 stage (p<0.05), N0 vs N2 stage (p<0.01). Furthermore, multivariate analysis revealed that HMGA1 was an independent risk factor of OS and DSS for LUAD patients (p<0.05). HMGA1 were positively correlated with glycolysis gluconeogenesis pathway and glycolysis markers (HK2, GLUT1, PKM2, LDHA) based on GSEA and Gene Expression Profiling Interactive Analysis (GEPIA) database. At the cellular level, the results of qRT-PCR and western blot assays showed that si-HMGA1 markedly decreased the expression of glycolysis markers. HMGA1 promoted cell glycolysis progression via PI3K/AKT pathway transfected with HMGA1-plasmid and the treatment with 20 μM LY294002. Relevant animal experiments were also synchronously validated and si-HMGA1 groups down-regulated xenograft growth including the weights and size in tumor xenografts.

**Conclusions:** In conclusion, our results suggested that HMGA1 was significantly correlated with poor survival for LUAD tissues and involved in the process of glycolysis in LUAD cells**.**

## Introduction

Lung cancer is one of the leading causes with high morbidity and mortality 1. Among them, approximately more than 85% of those cases are currently considered as non-small-cell lung cancer (NSCLC) and lung adenocarcinoma (LUAD) is the most common classification 2, 3. However, despite knowledge gains in tumor biology that have greatly advanced in recent decades, mortality from lung cancer remains high for most patients 4. Therefore, it is urgent to explore more further effective biomarkers for pathology diagnosis and prognosis prediction in LUAD.

High mobility group A (HMGA) proteins are small nuclear proteins with high mobility 5. HMGA1 serves as an architectural transcription factor to participate in the process of restructure of chromatin structure, which promotes the interaction between transcriptional regulatory proteins and DNA in various types of tumors 6. As a member of the HMGA family, HMGA1 has an important role in tumorigenesis and tumor progression among gastric cancer 7, breast cancer 8, colorectal cancer 9, ovarian cancer 10, thyroid cancer 11 and bladder cancer 12. However, the role and clinical significance of HMGA1in LUAD have not been frequently published so far. Our past research focused on the regulatory effect between microRNA-4458 and HMGA1 in lung cancer cells 13. Furthermore, our team in this study seeks to explore the specific role of HMGA1 in prognostic value and glycolysis progression based on The Cancer Genome Atlas (TCGA) database of LUAD.

In this research, we compared HMGA1 mRNA expression between tumor tissues and normal samples and evaluated the correlations with clinical characteristics in LUAD patients based on the data of TCGA database. The survival outcome with overall survival (OS), disease-specific survival (DSS) and clinicopathologic characteristics associated were performed using the Kaplan-Meier method and Cox regression. In addition, gene-set enrichment analysis (GSEA) was carried out to explore the biological pathways that related to HMGA1, which refer to the correlation of glycolysis gluconeogenesis pathway. Furthermore, cell experiments including cell proliferation assay and glycolysis proteins (HK2, GLUT1, LDHA, PKM2) were performed with A549 and H1299 cells. Regarding the molecular mechanism of HMGA1 regulating glycolysis in lung adenocarcinoma, we conducted the related experiments of PI3K/AKT pathway. PI3K/AKT signaling pathway plays an important role in the progression of tumor cells. Furtherly, after the treatment with LY294002 (an inhibitor of PI3K/AKT pathway; 20 μM), the results indicated that HMGA1 promoted cell glycolysis progression via PI3K/AKT pathway. At the same time, relevant animal experiments were also synchronously validated. The results proved that si-HMGA1 group inhibited xenograft growth including the weights and size in tumor xenografts. Our results demonstrated that HMGA1 was a potential diagnostic and prognostic biomarker and involved in the progression of glycolysis in LUAD.

## Materials and Methods

### Patient data sets

All RNA-seq data of HMGA1 expression and relevant clinical information were downloaded from TCGA database (https://portal.gdc.cancer.gov/) including 594 samples with workflow type of HTSeq-FPKM. Our team downloaded TPM format RNA-seq data of TCGA database and Genotype-Tissue Expression (GTEx) database from UCSC Xena 14 ( https://xenabrowser.net/datapages/) by toil progression. Inclusion criteria :1) Patients confirmed by lung adenocarcinoma pathology; 2) Patients without serious complications. Exclusion criteria:1) Patients with combined malignant tumors of other organs; 2) Patients whose dependency check cannot cooperate with follow-up.

### The GEO database and the human protein atlas

Microarray series of GSE27262 and GSE63459 were downloaded from the Gene Expression Omnibus (GEO, https://www.ncbi.nlm.nih.gov/geo/) database. GSE27262 dataset contained tumor and adjacent tissue pairs of 25 LUAD patients. GSE63459 is a microarray dataset that included 31 LUAD patients and 31 normal individuals. The Human Protein Atlas (HPA, https://proteinatlas.org/) is the tool to obtain the proteomic and transcriptome information of all the human proteins in the terms of cell, tissue and pathology atlas. Here, we performed HPA to confirm the protein expression of HMGA1 in lung adenocarcinoma and normal tissue.

### UALCAN database and CPTAC analysis

UALCAN (http://ualcan.path.uab.edu/) is a comprehensive and interactive web resource for analyzing cancer OMICS data. Furthermore, Clinical Proteomic Tumor Analysis Consortium (CPTAC) analysis were used to obtain the protein expression of HMGA1 in LUAD.

### HMGA1-interaction proteins and function and pathway enrichment analyses in LUAD

In order to predict the function of HMGA1, we used TCGA data to select Top 50 genes most negatively associated with HMGA1 in LUAD and the results were shown by the heatmap. Similarly, the top 300 genes most positively associated with HMGA1 in LUAD were selected out to obtain the information of function and pathway enrichment analyses. The Gene Ontology (GO) terms include biological processes (BP), molecular function (MF) and cell component (CC).

### Gene set enrichment analysis (GSEA) and Protein-protein interaction comprehensive analysis

GSEA is a computational method that determines whether concordant differences in two biological states are statistically significant. GSEA was conducted to identify potential biological pathways and processes between high-HMGA1 and low-HMGA1 groups. Gene set permutations were repeated 1000 times for each analysis. Normal p-value<0.05 and false discovery rate (FDR) q values<0.25 are considered to be statistically significant.

The Search Tool for the Retrieval of Interacting Genes/Proteins (STRING) website (https://string-db.org/) was used to carry on the protein-protein interaction (PPI) comprehensive analysis of HMGA1-binding proteins with the basic settings of low confidence (0.150) and no more than 50 interactors.

### Correlation analysis in Gene Expression Profiling Interactive Analysis (GEPIA) database

GEPIA database (http://gepia.cancer-pku.cn/index.html) is a multifunctional comprehensive gene expression analysis tool. Here we used GEPIA to obtain the correlation between HMGA1 and glycolysis markers (HK2, GLUT1, PKM2, LDHA) in LUAD. The statistical significance is dependent on p-value<0.05.

### Immune cells infiltration analysis by ssGSEA

The immune infiltration analysis of LUAD was conducted by single-sample gene set enrichment analysis (ssGSEA) 15, 16 method from GSVA R package (http://www.bioconductor.org/packages/release/bioc/html/GSVA.html) and the infiltration levels of 24 immune cell types were quantified from gene expression profile. In addition, the correlation analysis was performed by a Spearman method. P-value were determined by the Spearman and Wilcoxon rank sum test. The scatter diagram and group comparison diagram were performed to confirm the correlation of HMGA1 with Th2 cells and CD56dim cells.

### Cell transfection and cell proliferation analysis

The si-HMGA1 reagent (RiboBio, Guangzhou, China) was used for the knockdown of HMGA1 in A549 and H1299 cell lines. Transfection of cells was performed by riboFECT™ CP Transfection Agent following the manufacturer's protocol. Cell proliferation assay was assessed by CCK-8 assay (Dojindo, Kumamoto, Japan) and colony formation assay. After 24 hours of transfection, cells were seeded into 96-well plates with the amount of 2×10^3^ cells per well. Then the microplate reader (Thermo Fisher Scientific) was conducted at 450 nm wavelength. For Colony assay, cells transfected were seeded into 6-well plates with the amount of 200 cells per well for 14 days in cell incubator (37°C, 5% CO_2_). Then the cells were fixed in 70% ethanol and stained by crystal violet.

### Cell metabolic assays

The lactate production assays were measured by lactate assay kit (KeyGen) to detect the impact on glucose metabolites after transfection for 48h. The ATP assay kit (Beyotime) were used to conduct ATP production assay. For ^18^F-FDG uptake assay, the culture conditions are sugar free medium with the addition of one microcurie of ^18^F-FDG in cells. Afterwards, the cells are cultured in the incubator for 2 hours. The γ-counter were conducted to determine ^18^F-FDG radioactivity in the supernatant and the cells.

### qRT-PCR and western blotting assay

For qRT-PCR assay, the total RNA was extracted by TRIzol (Thermo Fisher Scientific, Waltham, MA, USA). The mRNA expression level was assessed by the SYBR Premix Ex Taq II kit (TaKaRa, Dalian, China) and then evaluated by the machine of Light Cycler 480 System II (Roche). For western blotting analysis, antibodies against HK2, GLUT1, LDHA, PKM2, HMGA1 were obtained from Abcam (Cambridge, UK) and while β-actin antibody served as an internal control. After the processes of lysis, separation of protein and transfer with transfected cells, the antibodies were incubated at 4°C overnight. The results of specific protein bands were analyzed by software of the Image Lab.

### The establishment of lung adenocarcinoma mouse xenograft

Twenty 4~5-week-old female balb/c nude mice were purchased from Beijing Vital River Laboratory Animal Technology and raised in the environment of SPF conditions. 2×10^7^ A549 cells were injeted into the right underarm dorsal side of mouse xenograft. Tumor volume formula is as follows: Tumor Volume= length×(width)^2^×0.5. The experimental animals were divided into two groups, si-HMGA1 group and si-HMGA1 NC group. The two groups of mouse xenograft were injected with si-HMGA1 or si-HMGA1 NC for 2weeks (5nmol per mouse, 3 times per week). Finally, mice were weighed, euthanized, and tumors were dissected for subsequent experiments.

### Statistical analysis

All statistical analysis at the cellular level was conducted by the software of GraphPad Prism 6.0 software (La Jolla, CA, USA). Outcomes were indicated with the mean ± SD among triplicate samples. All statistical analyses with bioinformatics were performed with R (Version 3.6.3). The Wilcoxon rank sum tests and Wilcoxon signed-rank tests were conducted to analyze the expression between LUAD and normal groups. The relationship between HMGA1 and clinicopathologic characteristics were carried out by the Wilcoxon signed-rank test and logistic regression. The survival outcome with OS, DSS and clinicopathologic characteristics associated were performed using the Kaplan-Meier method and Cox regression. P-value less than 0.05 was considered as statistically significant. The median expression value of HMGA1 was determined to be the cut-off value.

## Results

### Clinical features of the LUAD patients

A total of 594 patients including 535 primary tumors and 59 normal samples with clinical and gene expression data were acquired from TCGA database. The detailed clinical information includes Topography distribution (T) stage, Lymph node metastasis (N) stage, Distant metastasis (M) stage, Pathologic stage, Gender, Age, Smoker, OS event, DSS event and progression-free interval (PFI) event (Table 1).

### High expression and diagnostic value of HMGA1in LUAD patients

**Figure 1:**
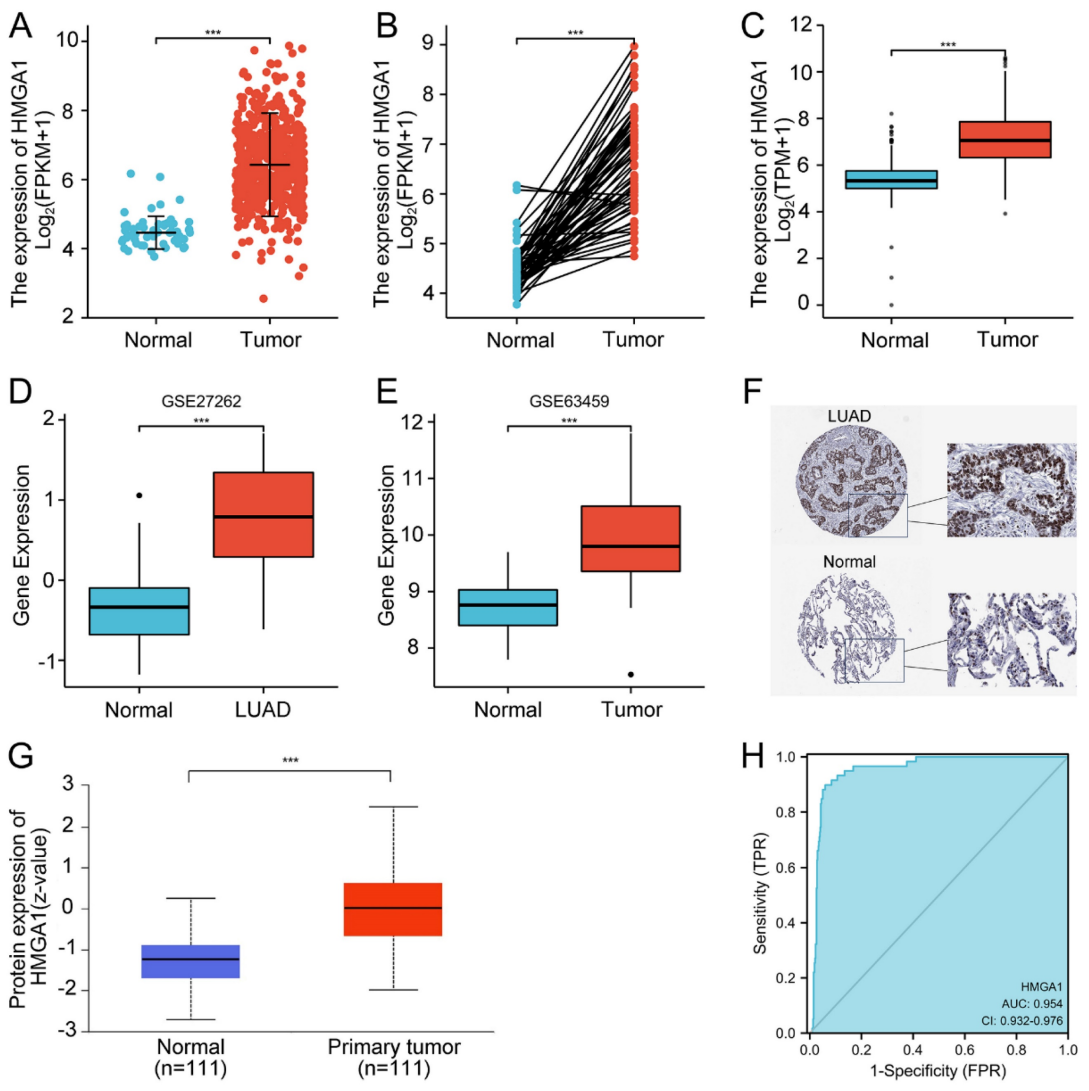
HMGA1 expression and ROC curve in LUAD tissues. (A) HMGA1 mRNA expression in LUAD tissues and normal tissues (p<0.001). (B) HMGA1 mRNA expression in LUAD tissues and adjacent tissues (p<0.001). (C) HMGA1 mRNA expression in LUAD tissues of TCGA and normal lung tissues of GTEx combined with TCGA (p<0.001). (D) The expression of HMGA1 in LUAD tissues from GSE27262 dataset (p<0.001). (E) The expression of HMGA1 in LUAD tissues from GSE63459 dataset (p<0.001). (F) The protein expression level of HMGA1 from the HPA database. (G) The protein expression of HMGA1 from UALCAN database by CPTAC analysis (p<0.001). (H) ROC curve of HMGA1 mRNA expression in LUAD tissues and normal tissues. *** p<0.001.

In order to assess the expression of HMGA1 in LUAD, we compared the expression level of LUAD patients with normal lung tissues from TCGA. The results showed that HMGA1 mRNA expression was significantly elevated in LUAD tissues than that in normal tissues (p<0.001, Figure 1A). Similar trend was also obtained in the expression of HMGA1in 57 LUAD tissues and paired normal lung tissues (p<0.001, Figure 1B). In addition, the expression of HMGA1 in normal samples of GTEx and TCGA compared with LUAD tissues of TCGA database was obtained, which demonstrated that HMGA1 was significantly overexpressed in LUAD samples (p<0.001, Figure 1C). Consistent with it, the upregulated HMGA1 in LUAD tissues was shown in the GSE27262 and GSE63459 datasets. (p<0.001, Figure 1D, E).

From the HPA database, we compared the expression of HMGA1 in the protein level using the IHC staining. The protein expression level of HMGA1 in LUAD tissues was higher than normal tissues (Figure 1F). In addition, we used the CPTAC analysis from UALCAN database to obtain the information of the protein expression of HMGA1. The results showed that the higher protein expression of HMGA1 in LUAD than that in normal tissues (p<0.001, Figure 1G). In the end, ROC curve was conducted to evaluate the diagnostic value of HMGA1. The results showed that the area under the curve (AUC) of HMGA1 was 0.954 (Figure 1H).

### Correlation between HMGA1 expression and clinical features

**Figure 2:**
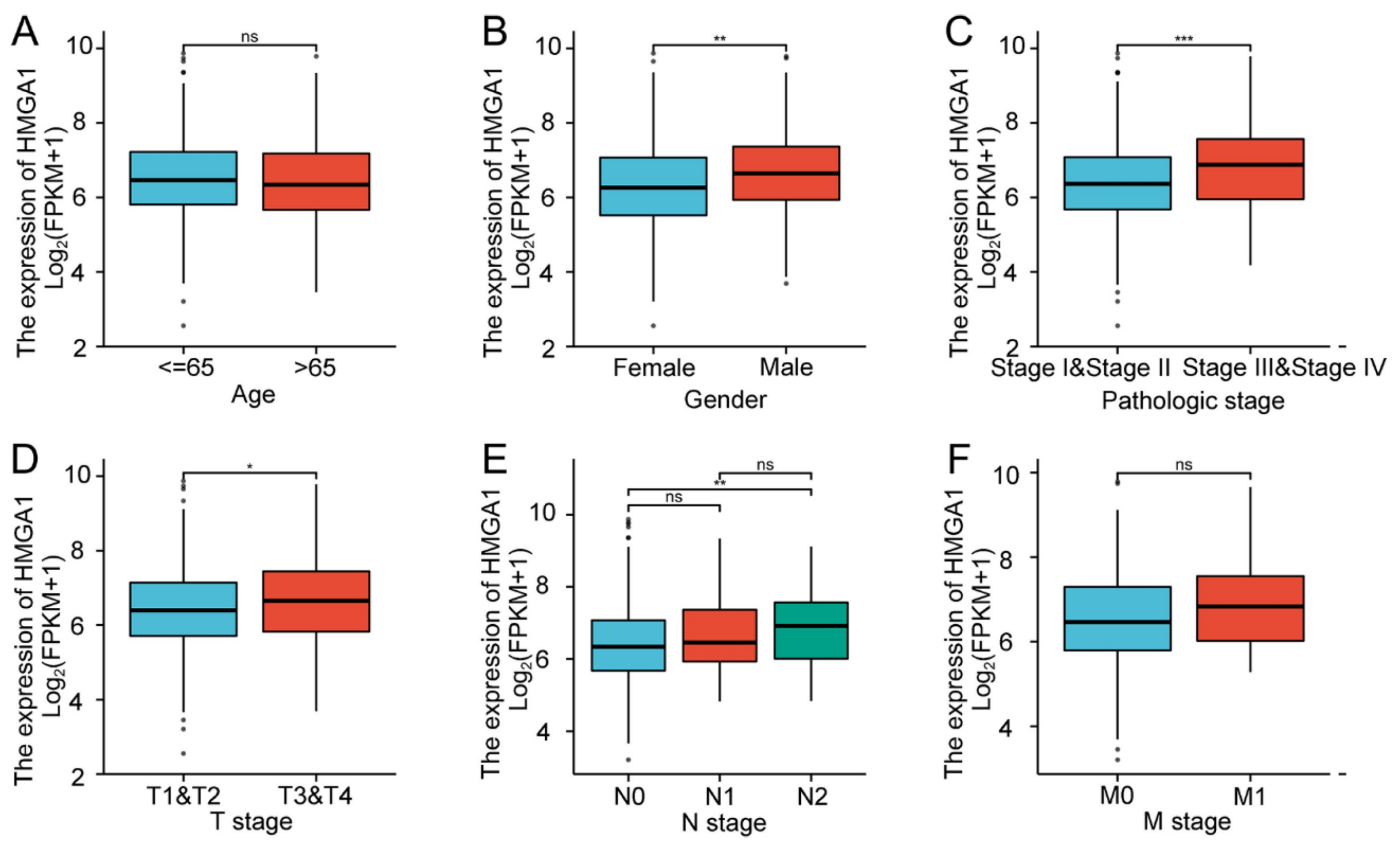
Association with HMGA1 expression and clinicopathologic characteristics in LUAD tissues. Notes: (A) Age. (B) Gender (p<0.01). (C)Pathologic stage (p<0.001). (D) T stage (T1&T2 vs T3&T4, p<0.05). (E) N stage (N0 vs N2, p<0.001). (F) M stage. * p<0.05, ** p<0.01, *** p<0.001.

In order to verify the association with HMGA1 expression and clinicopathologic characteristics in LUAD tissues, the box diagrams were conducted including the levels of Age, Gender, Pathologic stage, T stage, N stage and M stage (Figure 2). High expression of HMGA1 was significantly correlated with Gender (p<0.01), Pathologic stage I&II vs stage III&IV (p<0.001), T1&T2 vs T3&T4 stage (p<0.05), N0 vs N2 stage (p<0.01). However, other clinical features were not significantly correlated with high expression of HMGA1. In addition, more detailed information of the association between HMGA1 expression and clinical features including Smoker, OS event, DSS event and PFI event was shown in Table 2. The results indicated that elevated expression of HMGA1 was significantly correlated with Smoker (p<0.01), OS event (p<0.01), DSS event (p<0.05).

### HMGA1 expression is correlated with poor prognosis clinical features in LUAD tissues

**Figure 3:**
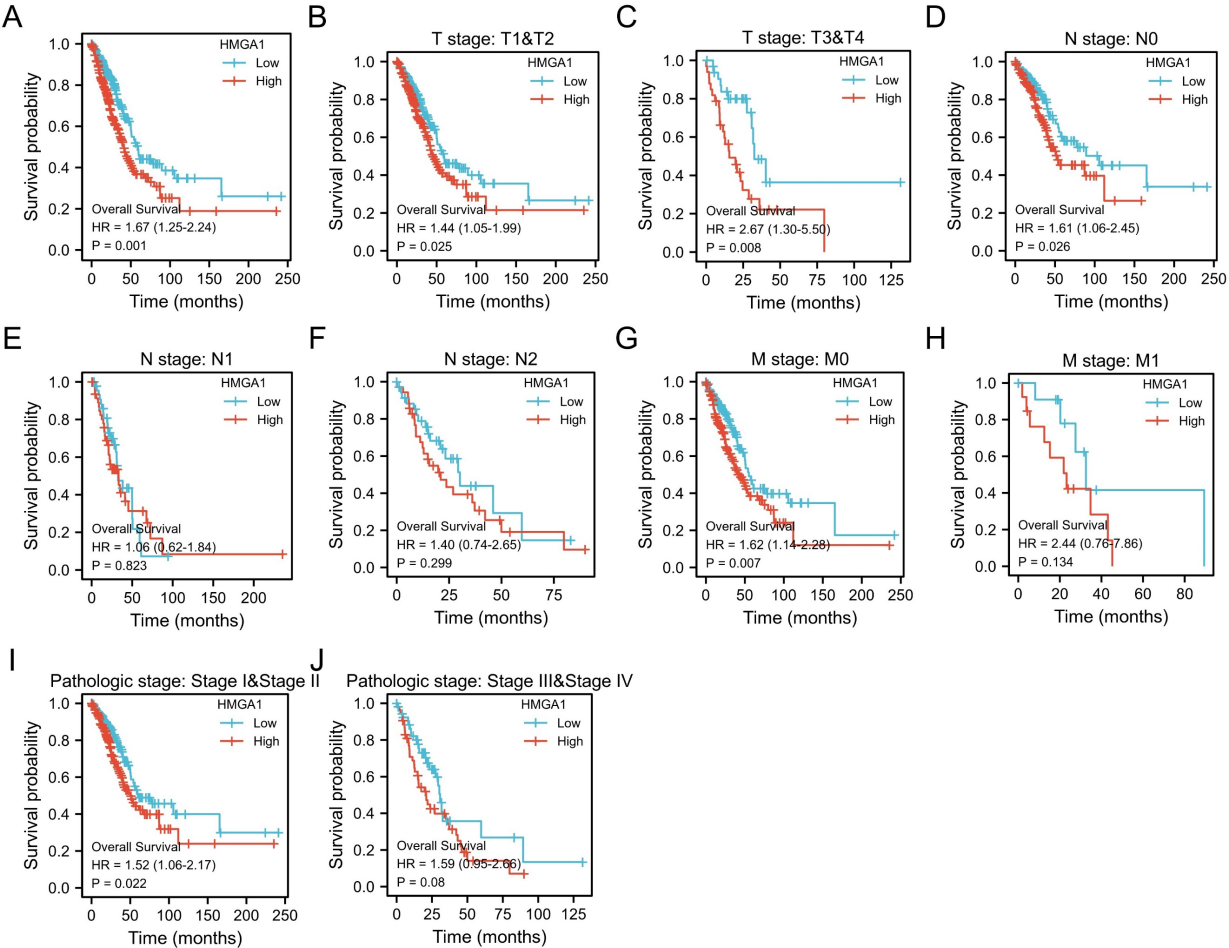
Kaplan-Meier curve of HMGA1 mRNA expression for overall survival in LUAD (A) Kaplan-Meier curve in all cases (p=0.001). (B) T1&T2 (p=0.025). (C) T3&T4 (p=0.008). (D) N0 (p=0.026). (E) N1 (p=0.823). (F) N2 (p=0.299). (G)M0 (p=0.007). (H) M1 (p=0.134). (I) stage I&II (p=0.022). (J) stage III&IV (p=0.08).

The Kaplan-Meier analysis was carried out to obtain the association between HMGA1 expression and OS of patients in LUAD. The results showed HMGA1 expression is positively correlated with poor OS in LUAD patients (p=0.001, Figure 3A). For subgroups analysis, the clinical features terms including T1&T2 (p=0.025), T3&T4 (p=0.008), N0 (p=0.026), M0 (p=0.007), stage I&II (p=0.022) indicated that high HMGA2 expression was significantly associated with poor prognosis in LUAD (Figure 3B-3J).

Univariate analysis using logistic regression demonstrated that HMGA1 expression was a categorical dependent variable associated with poor prognostic in LUAD (Table 3). High expression of HMGA1 was significantly associated with N stage (N2&N3 vs N0&N1, OR=1.948, 95% confidence interval [CI]=1.182-3.269, p=0.01), Gender (Male vs Female, OR=1.781, 95% CI=1.256-2.534, p=0.001), Pathologic stage (Stage III & Stage IV vs Stage I & Stage II, OR=1.733, 95%CI=1.130-2.681, p=0.012).

### Cox univariate and multivariate analysis of OS and DSS with HMGA1 expression in LUAD

The univariate Cox analysis revealed that high HMGA1 expression (Low vs High, p=0.03), T (T3&T4 vs T1&T2, p<0.001), N (N2&N3 vs N0&N1, p<0.001), M (M1 vs M0, p=0.007), Pathologic stage (S III&S IV vs S I&S II, p<0.001) were correlated with OS. Multivariate analysis showed that HMGA1 mRNA expression was an independent risk factor for OS in LUAD tissues (95% CI=1.113-2.277, p=0.011, Table 4).

For the univariate Cox analysis of DSS, the variables with p<0.05 were HMGA1 expression (Low vs High, p=0.013), T (T3&T4 vs T1&T2, p=0.006), N (N2&N3 vs N0&N1, p=0.004), M (M1 vs M0, p=0.007), Pathologic stage (SIII&SIV vs SI&SII, p<0.001). Multivariate analysis showed that HMGA1 mRNA expression was an independent risk factor for DSS in LUAD tissues (95% CI=1.086-2.766, p=0.021, Table 5).

### HMGA1-interaction proteins and function and pathway enrichment analyses in LUAD

**Figure 4:**
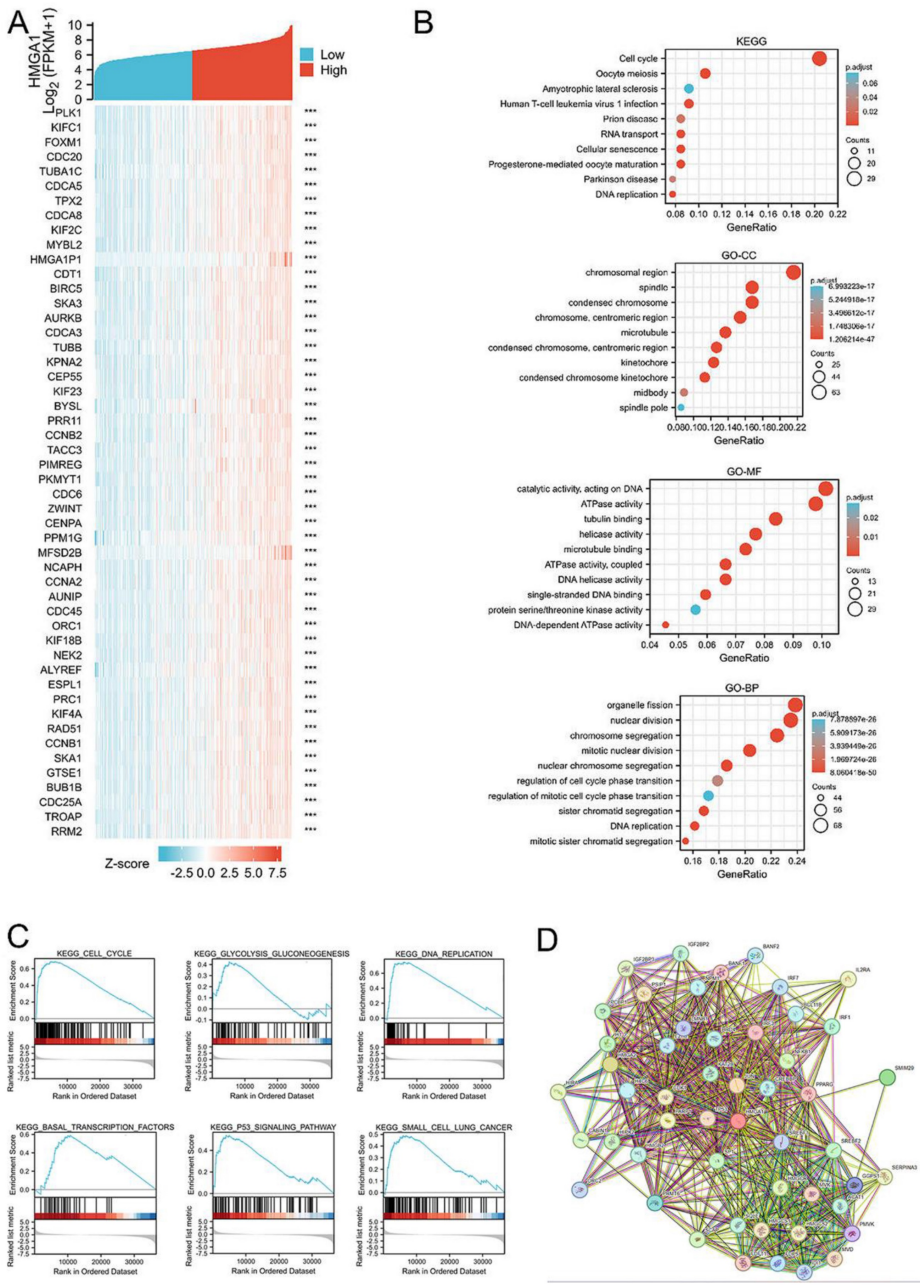
HMGA1-related gene enrichment analysis. (A) The heatmap of top 50 genes most negatively associated with HMGA1 using TCGA data. (B) Function and pathway enrichment analyses of the top 300 genes most positively associated with HMGA1 in LUAD. (C) Enrichment plots from GSEA with high HMGA1 expression in LUAD. (D) Protein-protein interaction comprehensive analysis of HMGA1-binding proteins using the STRING tool.

Correlation analysis between HMGA1 and other mRNAs in LUAD was carried out by TCGA data, with the analysis of pearson correlation coefficient. The corresponding heatmap data in the detailed of top 50 genes most negatively associated with HMGA1 are shown (Figure 4A).

Similarly, the top 300 genes most positively associated with HMGA1 in LUAD were selected out to obtain the information of function and pathway enrichment analyses. The Gene Ontology (GO) terms include biological processes (BP), molecular function (MF) and cell component (CC). KEGG pathway analysis indicated that HMGA1 was primarily associated with cell cycle and oocyte meiosis. In the terms of GO analysis, chromosomal region and spindle, catalytic activity, acting on DNA and ATPase activity, organelle fission and nuclear division was associated with HMGA1(Figure 4B).

### Protein-protein interaction comprehensive analysis

STRING tool was used to analyze the PPI network of HMGA1 protein to determine their interactions in the progression of LUAD. The top 10 proteins and corresponding gene included: C6orf1, CEBPB, EP400, HMGA1, HMGA2, HMGCR, INSIG1, LMNB1, RB1, RPS6KB1 (Figure 4D).

### GSEA identifies a HMGA1-related signaling pathway

GSEA database were performed to identify the signaling pathway correlated with HMGA1 expression. NOM p value<0.05, FDR q value<0.25 were considered as reference standard. From the enrichment of signaling pathways, we selected pathways related to six biological processes, including cell cycle, glycolysis gluconeogenesis, DNA replication, basal transcription factors, p53 signaling pathway and small cell lung cancer (Figure 4C). The more detailed information about gene sets enriched in the high expression of HMGA1 were shown in the Table 6.

### Correlation analysis with glycolysis markers based GEPIA

**Figure 5:**
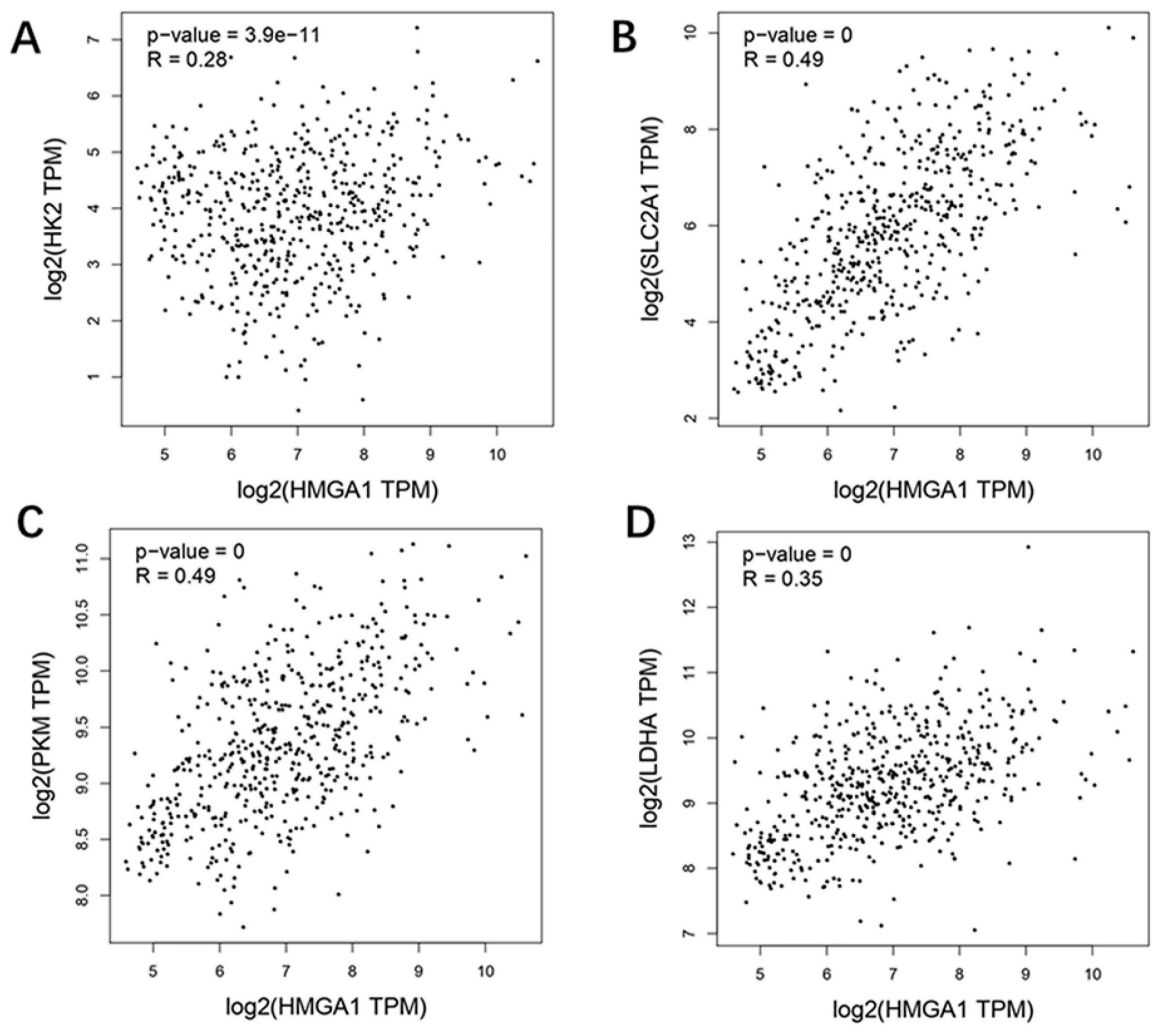
Correlation analysis between HMGA1 and glycolysis markers (HK2, SLC2A1[GLUT1] PKM2, LDHA) in LUAD via GEPIA database. (A) HK2. (B) GLUT1. (C) PKM2. (D) LDHA.

GEPIA database was carried out to obtain the analysis of correlation between HMGA1 and glycolysis markers (HK2, SLC2A1[GLUT1], PKM2, LDHA) in LUAD. The results showed that four glycolysis markers including HK2 (R=0.28, p-value=3.9e-11), GLUT1 (R=0.49, p-value=0), PKM2 (R=0.49, p-value=0), LDHA (R=0.35, p-value=0) were positively correlated with HMGA1 expression (Figure 5).

### HMGA1 promoted cell proliferation and regulated cell glycolysis progression of LUAD cells via PI3K/AKT pathway

**Figure 6:**
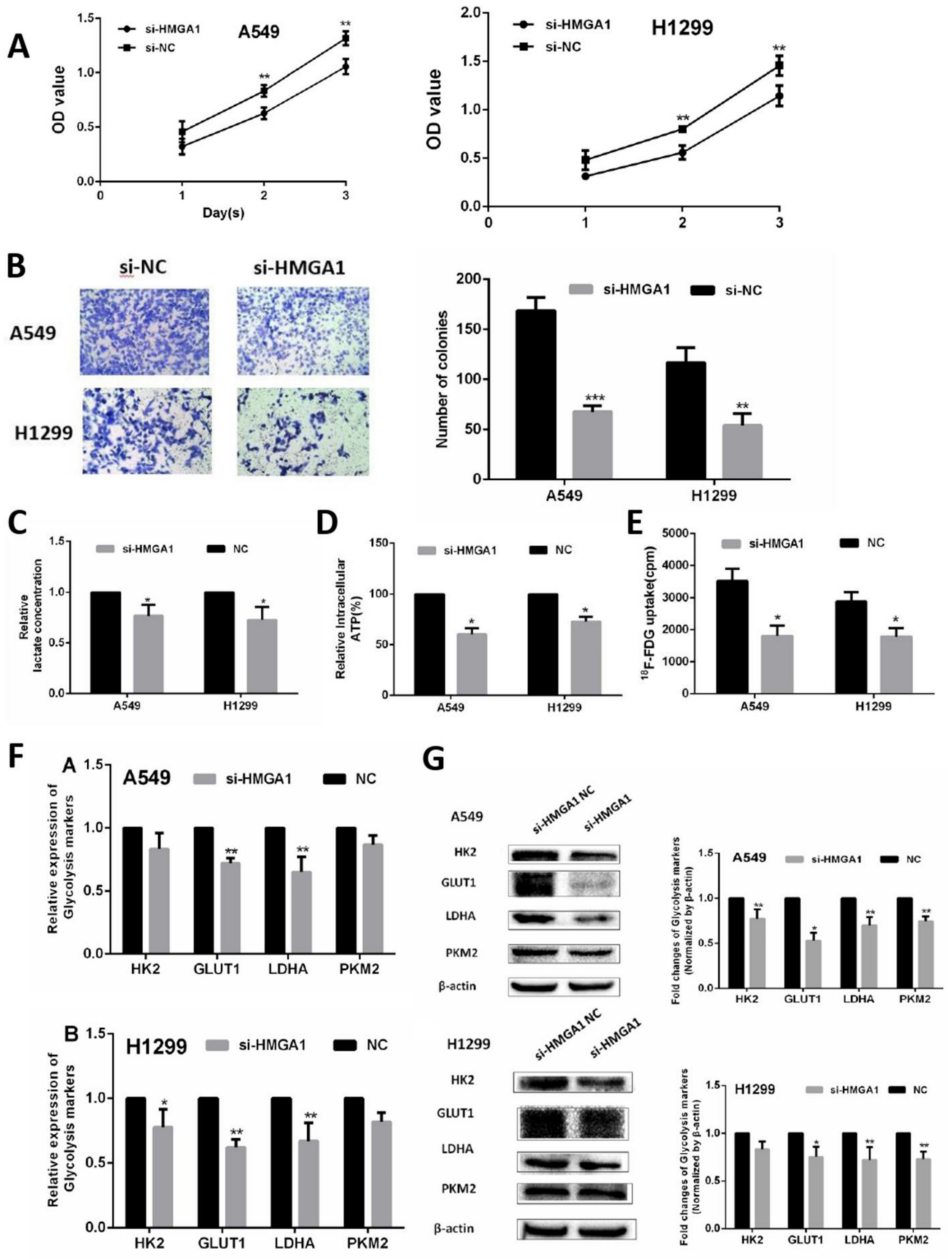
HMGA1 promoted cell proliferation and regulated cell glycolysis progression in LUAD cells and in vivo. (A) CCK-8 assays were conducted in A549 cell and H1299 cell transfected with si-HMGA1. (B) Colony assays were performed transfected with si-HMGA1. (C) Lactate production assay. (D) ATP production assay. (E) ^18^F-FDG uptake assay. (F) The RNA expression of glycolysis markers (HK2, GLUT1, PKM2, LDHA) were confirmed by qRT-PCR transfected with si-HMGA1. (G) The protein expression of glycolysis markers (HK2, GLUT1, PKM2, LDHA) were confirmed by western blot assay transfected with si-HMGA1.

In the terms of cell proliferation, CCK-8 assays and colony assays were conducted in A549 cell and H1299 cell transfected with siRNA (si)-HMGA1 and si-NC. The results of two assays indicated that HMGA1 knockdown markedly inhibited the proliferation ability of LUAD cells (Figure 6A, 6B, p<0.01). To verify the regulation of glycolysis progression, cell metabolic assays were conducted by assay kit and the γ-counter. The lactate production assay and ATP production assay were measured by lactate assay kit and ATP assay kit after transfection for 48h. The results showed that the knockdown of HMGA1 inhibited the lactate production and ATP production (Figure 6C, 6D, p<0.05). For ^18^F-FDG uptake assay, the γ-counter were conducted to determine ^18^F-FDG radioactivity in the supernatant and the cells. The results informed that ^18^F-FDG uptake decreased after transfection with si-HMGA1 compared with NC group (Figure 6E, p<0.05).

**Figure 7:**
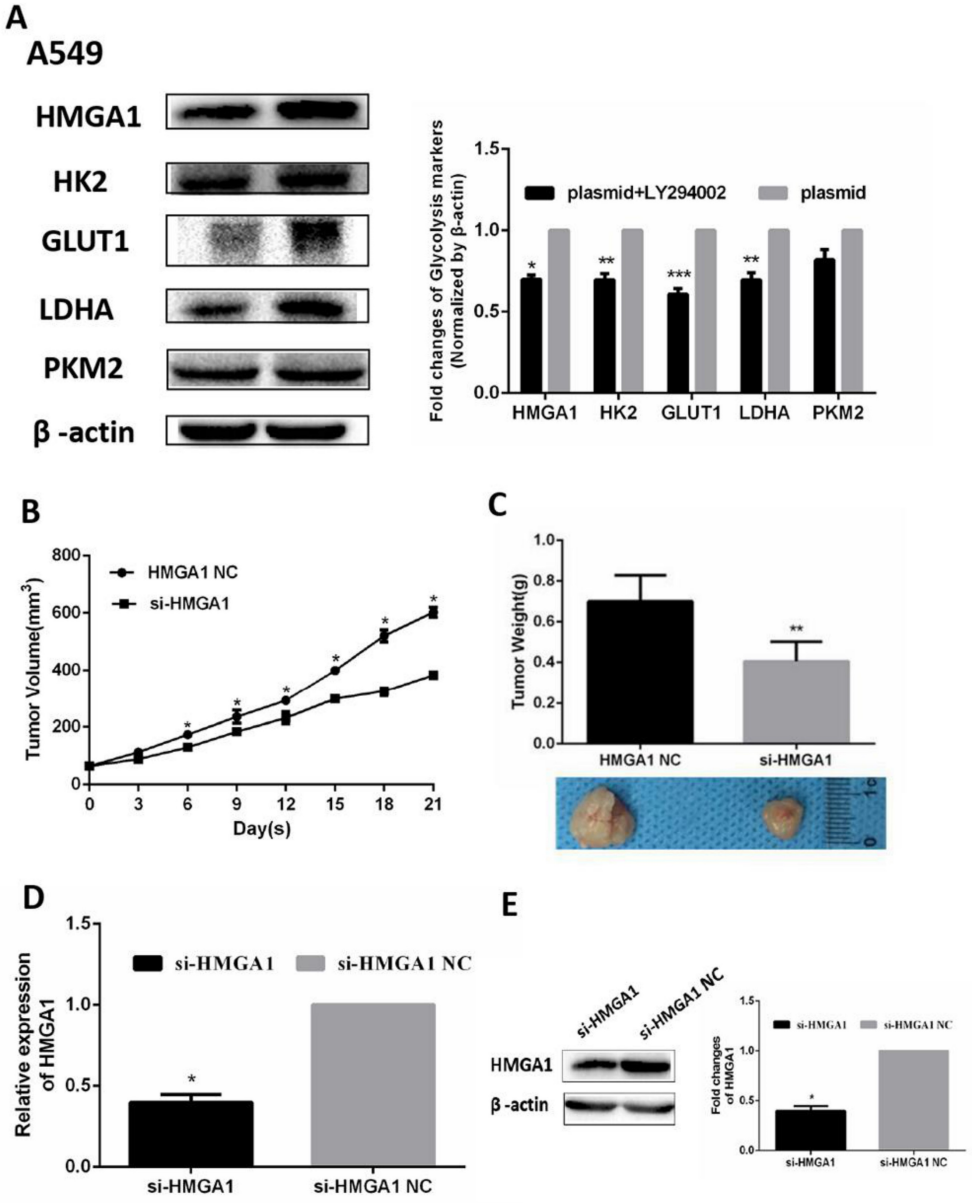
HMGA1 promoted cell glycolysis progression via PI3K/AKT pathway and HMGA1 regulated xenograft growth. (A) The protein expression of glycolysis markers (HK2, GLUT1, PKM2, LDHA) were confirmed by western blot assay transfected with HMGA1-plasmid and the treatment with 20 µM LY294002. (B)The growth curve of tumor xenograft volumes. (C) The weights and comparison of size in tumor xenografts. (D) The levels of HMGA1 were performed by qRT-PCR. (E) The levels of HMGA1 were performed by western blot assay.

In addition, the RNA expression of relevant glycolysis markers including HK2, GLUT1, PKM2, LDHA were determined qRT-PCR. The results demonstrated HMGA1 knockdown decreased the expression of glycolysis markers (Figure 6F, p<0.05). In addition, western blot assays were carried out to explain the protein expression of glycolysis markers. The results showed that si-HMGA1 markedly decreased the expression of glycolysis markers (Figure 6G, p<0.05). Regarding the molecular mechanism of HMGA1 regulating glycolysis in LUAD cells, the related experiments of PI3K/AKT pathway were conducted in the A549 cell. After the treatment with LY294002 (an inhibitor of PI3K/AKT pathway; 20 μM), the protein expression of glycolysis markers (GLUT1, HK2, PKM2, LDHA) were confirmed by western blot assay. The results indicated that HMGA1 promoted cell glycolysis progression of LUAD cells via PI3K/AKT pathway (Figure 7A, p<0.05).

### The establishment of lung adenocarcinoma mouse xenograft

A549 tumor mouse xenografts were constructed by injection with si-HMGA1 or si-HMGA1 NC. The growth curves of tumor xenograft volumes were significantly reduced in the si-HMGA1 group (Figure 7B, p<0.05). The weights and comparison of size in tumor xenografts were shown in Figure 7C. The levels of HMGA1 were performed by qRT-PCR and western blot assay. The results informed that the expression of HMGA1 was lower in the si-HMGA1 group than that in the NC group (Figure 7D, 7E, p<0.05).

### Immune infiltration analysis of HMGA1 in LUAD

**Figure 8:**
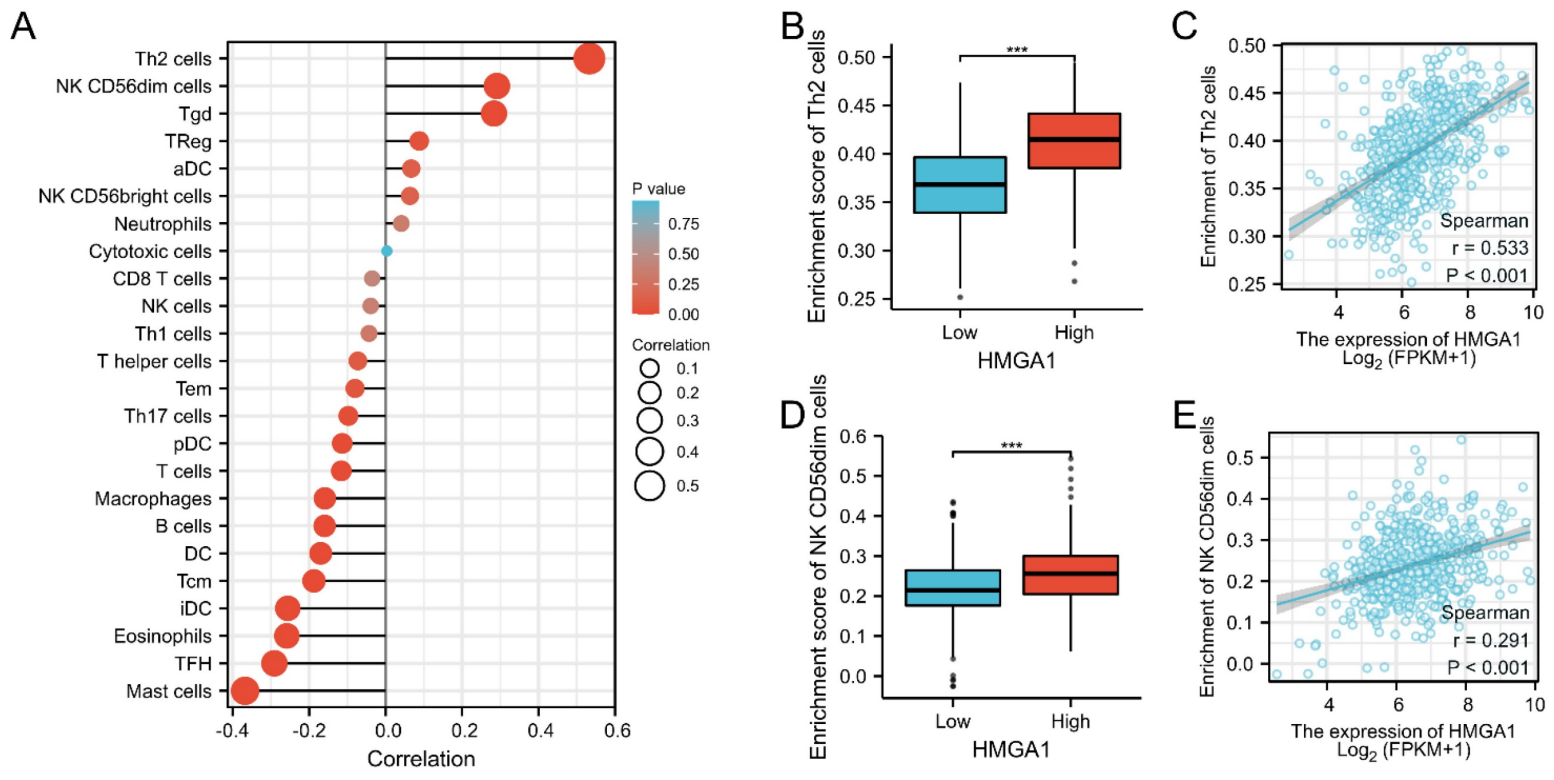
Immune infiltration analysis of HMGA1 in LUAD. (A) The correlation between HMHGA1 expression and 24 immune cells was shown by forest plot. (B)The correlation between HMHGA1 expression and Th2 cells by box diagram. (C) The correlation between HMHGA1 expression and Th2 cells by scatter plot. (D) The correlation between HMHGA1 expression and CD56dim cells by box diagram (E) The correlation between HMHGA1 expression and CD56dim cells by scatter plot. *** p<0.001.

We further analyzed the correlation between expression of HMGA1 and immune infiltration by ssGSEA. Firstly, the correlation analysis of HMGA1 expression and 24 immune cells was shown by forest plot (Figure 8A). Further, we selected Th2 cells to display the detailed information of the correlation by box diagram and scatter plot (Figure 8B, 8C, p<0.001). Similarly, CD56dim cells were used to analysis the relationship with HMGA1 expression by the scatter diagram and group comparison diagram (Figure 8D, 8E, p<0.001).

## Discussion

In this study, we used TCGA data to detect the expression of HMGA1 in LUAD and the correlation with diagnosis and prognosis. HMGA1 mRNA expression was higher in LUAD tissues than in normal tissues. Increased HMGA1 expression in LUAD was associated with Gender, Pathologic stage I&II vs stage III&IV, T1&T2 vs T3&T4 stage, N0 vs N2 stage. Furthermore, multivariate analysis revealed that HMGA1 was an independent risk factor of OS and DSS for LUAD patients. Previous studies have shown that HMGA1 plays a pivotal role in regulating the biological behavior of various tumors. HMGA1 has been reported to contribute to estrogen-independence, tumor progression, and poor outcomes and could serve as a prognostic marker and therapeutic target for breast cancer 17. For endometrioid endometrial carcinoma, one study shows that overexpression of HMGA1 figures as a potential prognostic factor 18. Another research indicates that the HMGA1 expression is significantly associated with a poor prognosis in esophageal squamous cell carcinoma patients, which suggests that downregulation of HMGA1 may become a practical treatment strategy 19. All these findings suggest the expression of HMGA1 play an important role in the survival of diagnosis and prognosis in various tumors.

HMGA1 has also participated in the regulation of long noncoding RNAs (lncRNAs), circular RNAs (circRNA) and microRNA (miRNA). Notably, our previous study conducts the further research on the regulation of HMGA1 and miR-4458, which suggests that miR-4458 participates in the process in NSCLC via targeting HMGA1 13. Other research of HMGA1 shows that HMGA1 has been identified as a target of miR-296-5p, which involves in cell proliferation in colorectal cancer 20. Additionally, ST8SIA6-AS1 contributes to hepatocellular carcinoma progression by targeting miR-142-3p/HMGA1 axis 21 and long non-coding RNA MYU promotes ovarian cancer cell proliferation by sponging miR-6827-5p and upregulating HMGA1 22. Interestingly, a recent study focuses on the terms of the deregulation of a putative cis-acting lncRNA in LUAD, and its effect on the oncogene HMGA1 23. The results suggest that HMGA1-lnc is a novel cis-acting lncRNA that negatively regulates HMGA1 gene expression in lung cells, which reminds us that HMGA1 plays an important role in the biological behavior of LUAD from different aspects.

As an important transcription factor, several researches have been conducted to focus on the further cellular mechanism. A recent study demonstrates that HMGA1 stimulates gastric cancer proliferation and metastasis via transactivating SUZ12 and CCDC43 expression 24. In B-cell neoplasms, one study indicates that HMGA1 binds to EZH2 promoter and sustains its transcription, which suggests a novel molecular mechanism contributing to EZH2 overexpression in human malignancies 25. In order to learn more about related biological activities with HMGA1, we firstly carried out GSEA database to explore six biological pathways that related to HMGA1 including cell cycle, glycolysis gluconeogenesis, DNA replication, basal transcription factors, p53 signaling pathway and small cell lung cancer. We selected glycolysis gluconeogenesis pathway to do further research. GEPIA database was carried out to obtain the analysis of correlation between HMGA1 and glycolysis markers (HK2, GLUT1, PKM2, LDHA) in LUAD. The results showed that four glycolysis markers were positively correlated with HMGA1 expression. Although various bioinformatics databases were learned about the function of HMGA1 in LUAD, we have conducted relevant basic experimental arrangement to further verify its role in different aspects. Firstly, the RNA expression of relevant glycolysis markers (HK2, GLUT1, PKM2, LDHA) were determined in A549 cell and H1299 cell transfected with si-HMGA1 by qRT-PCR. The results indicated that HMGA1 knockdown markedly inhibited the RNA expression of glycolysis markers. Then western blot assays were carried out to explain the protein expression of glycolysis markers and the results showed that si-HMGA1 markedly decreased the expression of glycolysis markers. The cellular assays indicated the active function on glycolysis of HMGA1 in LUAD. Regarding the molecular mechanism of HMGA1 regulating glycolysis in lung adenocarcinoma, we added the related experiments of PI3K/AKT pathway. PI3K/AKT signaling pathway plays an important role in the progression of tumor cells. Furtherly, after the treatment of HMGA1-plasmid and the treatment with LY294002 (an inhibitor of PI3K/AKT pathway; 20 μM), the results indicated that HMGA1 promoted cell glycolysis progression via PI3K/AKT pathway. Furthermore, animal experiments were conducted simultaneously. The results informed that the expression of HMGA1 was lower in the si-HMGA1 group than that in the NC group. Significantly, one study of colorectal cancer shows that HMGA1 promotes hepatic metastasis by inducing expression of glucose transporter 3 (GLUT3) 9. Similarly, HMGA1 promoting gastric cancer oncogenic and glycolytic phenotypes by regulating c-myc expression and identify a novel function of HMGA1 in regulating aerobic glycolysis in gastric cancer 26.

This article innovatively provides detailed mechanism and correlated impacts of HMGA1 in lung adenocarcinoma by bioinformatics. In clinical terms, the correlations with clinical characteristics, the prognostic value of HMGA1 and Kaplan-Meier analysis were conducted bases on TCGA database. The utilize of various bioinformatics software could present different aspects of data analysis of HMGA1. In order to verify the authenticity and reliability of the data, our research team applied cell and animal experiments for further verification. Above all, the combination of extensive experimental screening in bioinformatics and data validation in basic experiments makes the mechanism of HMGA1 in lung adenocarcinoma more authentic and reliable, which is also one of the creative points of this article. Cancer immunotherapy has changed the treatment landscape for cancer strategy and is gradually becoming the highlight of scientific research 27, 28. In our study, we have conducted a preliminary exploration of immune infiltration with HMGA1 in LUAD via bioinformatics analysis of ssGSEA. The correlation analysis of HMGA1 expression and 24 immune cells was shown by forest plot. From 24 immune cells, we selected Th2 cells and CD56dim cells to display the detailed information of the correlation by box diagram and scatter plot with P-value less than 0.001. Our study demonstrated that HMGA1 might participate in the immune infiltration process of tumors. Interestingly, one recent research indicates that HMGA1 stimulates MYH9-dependent ubiquitination of GSK-3β via PI3K/Akt/c-Jun signaling, which promotes malignant progression and chemoresistance in gliomas 29. Another recent review discusses the functional roles of ubiquitination in key T-cell activation to highlight the vast possibilities that targeting ubiquitination offers for advancing T-cell-based immunotherapies 30. The result of one study in colorectal cancer shows that PD-L1 expression is correlated with the expression of colorectal cancer stem cells markers and HMGA1 in clinical colorectal specimens 31. All above studies mentioned confirm that HMGA1 might play a certain biological role in immune infiltration. However, there are still shortcomings in our article. Firstly, we have only conducted preliminary basic research on the PI3K/AKT pathway through western blot transfected with HMGA1-plasmid and LY294002. Further in-depth research should be conducted on this aspect in the future. Secondly, we found that HMGA1 plays a role in the prognosis, proliferation, glycolysis and immunity of LUAD by bioinformatics and cell experiments. However, there is no specific mechanism research on this aspect so far. As an important target in LUAD, studying the specific roles and pathways of HMGA1, especially its regulatory role in immunity, will have certain clinical value and significance. Our team will gradually carry out the experimental study on the mechanism of immune infiltration in the future.

## Conclusions

In conclusion, our results suggested that HMGA1 was significantly correlated with poor survival for LUAD tissues and involved in the process of glycolysis in LUAD cells.

## Figures and Tables

**Table 1:** Clinical characteristics of the LUAD patients

Clinical characteristic	Variable	Overall	%
T stage	T1	168	32.9
	T2	276	54.1
	T3	47	9.2
	T4	19	3.7
N stage	N0	330	65.9
	N1	95	19
	N2	74	14.8
	N3	2	0.4
M stage	M0	344	93.2
	M1	25	6.8
Pathologic stage	Stage I	274	54.3
	Stage II	121	24
	Stage III	84	16.6
	Stage IV	26	5.1
Gender	Female	276	53.8
	Male	237	46.2
Age	<=65	238	48.2
	>65	256	51.8
Smoker	No	74	14.8
	Yes	425	85.2
OS event	Alive	326	63.5
	Dead	187	36.5
DSS event	Alive	362	75.9
	Dead	115	24.1
PFI event	Alive	304	59.3
	Dead	209	40.7

T, Topography distribution; N, Lymph node metastasis; M, Distant metastasis; OS, Overall survival; DSS, Disease-specific survival; PFI, Progression-free interval The number of missing information as follows: T stage (25), N stage (34), M stage (166), Pathologic stage (30), Gender (22), Age (41), Smoker (36), OS event (22), DSS event (58) and PFI event (22).

**Table 2:** Correlation between HMGA1 mRNA expression and clinicopathological characteristics in LUAD

Clinical Characteristic	Variable	Overall	HMGA1 mRNA		
			Low expression (%)	High expression (%)	*p* value
T stage	T1	168	100 (19%)	68 (13.3%)	0.022
	T2	276	125 (24%)	151 (29.6%)	
	T3	47	20 (3.9%)	27 (5.3%)	
	T4	19	10 (2%)	9 (1.8%)	
N stage	N0	330	173 (34.5%)	157 (31.3%)	0.040
	N1	95	47 (9.4%)	48 (9.6%)	
	N2	74	26 (5.2%)	48 (9.6%)	
	N3	2	1 (0.2%)	1 (0.2%)	
M stage	M0	344	165 (44.7%)	179 (48.5%)	0.574
	M1	25	10 (2.7%)	15 (4.1%)	
Pathologic stage	Stage I	274	150 (29.7%)	124 (24.6%)	0.045
	Stage II	121	58 (11.5%)	63 (12.5%)	
	Stage III	84	32 (6.3%)	52 (10.3%)	
	Stage IV	26	11 (2.2%)	15 (3%)	
Gender	Female	276	156 (30.4%)	120 (23.4%)	0.002
	Male	237	100 (19.5%)	137 (26.7%)	
Age	<=65	238	112 (22.7%)	126 (25.5%)	0.179
	>65	256	137 (27.7%)	119 (24.1%)	
Smoker	No	74	48 (9.6%)	26 (5.2%)	0.008
	Yes	425	201 (40.3%)	224 (44.9%)	
OS event	Alive	326	178 (34.7%)	148 (28.8%)	0.007
	Dead	187	78 (15.2%)	109 (21.2%)	
DSS event	Alive	362	193 (40.5%)	169 (35.4%)	0.027
	Dead	115	47 (9.9%)	68 (14.3%)	
PFI event	Alive	304	158 (30.8%)	146 (28.5%)	0.298
	Dead	209	98 (19.1%)	111 (21.6%)	

T, Topography distribution; N, Lymph node metastasis; M, Distant metastasis; OS, Overall survival; DSS, Disease-specific survival; PFI, Progression-free interval

**Table 3:** Relationship between HMGA1 expression and clinicopathologic characteristics using logistic analysis

Clinical Characteristics	Overall (N)	HMGA1 expression		
		OR	95%CI	*p* value
T stage (T3 & T4 vs T1 & T2)	510	1.233	0.735-2.082	0.429
N stage (N2 & N3 vs N0 & N1)	501	1.948	1.182-3.269	0.010
M stage (M1 vs M0)	369	1.383	0.611-3.261	0.443
Age (>65 vs <=65)	494	0.772	0.542-1.099	0.152
Gender (Male vs Female)	513	1.781	1.256-2.534	0.001
Pathologic stage (Stage III & Stage IV vs. Stage I & Stage II)	505	1.733	1.130-2.681	0.012

T, Topography distribution; N, Lymph node metastasis; M, Distant metastasis; OR, Odds ratio; CI, Confidence interval

**Table 4:** Association between os and clinicopathologic characteristics analyzed by univariate and multivariate Cox regression

Clinical Characteristics		Univariate analysis					Multivariate analysis		
	HR		95% CI	*p* value		HR		95% CI	*p* value
T (T3&T4 vs T1&T2)	2.364		1.621-3.448	<0.001		1.912		1.184-3.088	0.008
N (N2&N3 vs N0&N1)	2.300		1.614-3.278	<0.001		1.202		0.571-2.531	0.629
M (M1 vs M0)	2.111		1.232-3.616	0.007		1.207		0.537-2.712	0.649
Age (>65 vs <=65)	1.228		0.915-1.649	0.171					
Gender (Male vs Female)	1.060		0.792-1.418	0.694					
Pathologic stage (S III&S IV vs S I&S II)	2.624		1.926-3.576	<0.001		1.774		0.814-3.863	0.149
HMGA1 expression (Low vs High)	1.566		1.167-2.102	0.03		1.592		1.113-2.277	0.011
									

T, Topography distribution; N, Lymph node metastasis; M, Distant metastasis; HR, Hazard ratio; CI, Confidence interval

**Table 5:** Association between dss and clinicopathologic characteristics analyzed by univariate and multivariate Cox regression

Clinical Characteristics		Univariate analysis					Multivariate analysis		
	HR		95% CI	*p* value		HR		95% CI	*p* value
T (T3&T4 vs. T1&T2)	2.055		1.236-3.417	0.006		1.831		0.920-3.644	0.085
N (N2&N3 vs. N0&N1)	1.984		1.252-3.145	0.004		1.291		0.430-3.881	0.649
M (M1 vs. M0)	2.480		1.278-4.811	0.007		1.987		0.589-6.704	0.268
Age (>65 vs. <=65)	1.039		0.713-1.513	0.842					
Gender (Male vs. Female)	0.956		0.658-1.390	0.815					
Pathologic stage (S III&S IV vs. S I&S II)	2.443		1.643-3.632	<0.001		1.409		0.437-4.544	0.566
HMGA1 expression (Low vs High)	1.610		1.105-2.347	0.013		1.733		1.086-2.766	0.021
									

T, Topography distribution; N, Lymph node metastasis; M, Distant metastasis; HR, hazard ratio; CI, confidence interval

**Table 6:** Gene sets enriched in the phenotype with high HMGA1 expression

Gene set name	Set size	Enrichment score	NES	NOM *p* value	FDR *q* values	Rank	
BASAL_TRANSCRIPTION_FACTORS	35	0.590	1.900	0.008	0.071	10679	
DNA_REPLICATION	36	0.749	2.453	0.008	0.071	6096	
GLYCOLYSIS_GLUCONEOGENESIS	61	0.425	1.612	0.011	0.071	5168	
P53_SIGNALING_PATHWAY	68	0.540	2.039	0.013	0.071	3981	
SMALL_CELL_LUNG_CANCER	84	0.491	1.918	0.018	0.077	6776	
CELL_CYCLE	124	0.687	2.879	0.034	0.111	6301	

NES, Normalized enrichment score; NOM p value, Normal p-value; FDR q values, False discovery rate q values
